# Improvement in the long-term care burden after surgical treatment of patients with idiopathic normal pressure hydrocephalus: a supplementary study

**DOI:** 10.1038/s41598-021-90911-2

**Published:** 2021-06-03

**Authors:** Masatsune Ishikawa, Shigeki Yamada, Masakazu Miyajima, Hiroaki Kazui, Etsuro Mori

**Affiliations:** 1 Rakuwa Villa Ilios, Kyoto, Kyoto Japan; 2 Normal Pressure Hydrocephalus Centre, Otowa Hospital, Kyoto, Kyoto Japan; 3 Department of Neurosurgery, Shiga University of Medical Science, Otsu, Shiga Japan; 4 Department of Neurosurgery, Juntendo Tokyo Koto Geriatric Medical Center, Kotoku, Tokyo, Japan; 5 Department of Neuropsychiatry, Kochi Medical School, Kochi University, Nankoku, Kochi Japan; 6 Department of Behavioral Neurology and Neuropsychiatry, Osaka University United Graduate School of Child Development, Toyonaka, Osaka Japan

**Keywords:** Neurology, Surgery

## Abstract

Idiopathic normal pressure hydrocephalus (iNPH) is a surgically treatable syndrome commonly observed in older adults. However, it is unclear whether clinical improvements after surgery can effectively reduce the long-term care burden (LTCB). In this study, we determined whether shunt surgery was effective in decreasing LTCB. We also investigated the degree of variability in patients and hospitals, using data from the iNPH multicenter study. This study involved 69 participants who underwent lumboperitoneal shunt surgery with follow-up for 12 months. A generalized linear mixed model was applied to analyze the fixed and random effects simultaneously. Regarding LTCB, the disability grades improved significantly. Although the dementia grades also improved, it was not statistically significant. The differences in the LTCB grades in most patients were within the range of the 95% confidence intervals, while in the case of hospitals, some were often out of the range. Further studies are needed to improve dementia in patients with iNPH. The incorporation of random variables, such as hospitals, is important for the analysis of data from multicenter studies.

## Introduction

Idiopathic normal pressure hydrocephalus (iNPH) is a treatable syndrome that is commonly observed in older adults^1^. Cerebrospinal fluid (CSF) shunt surgery is an effective treatment for gait disturbance, dementia, and urinary incontinence, and it improves the symptoms and activities of daily living in patients with iNPH^2,3^. Our previous multicenter prospective studies on ventriculoperitoneal shunt surgery (study of idiopathic normal pressure hydrocephalus on neurological improvement: SINPHONI)^4^ and lumboperitoneal (LP) shunt surgery (SHINPHONI-2: SIN2)^5,6^ for iNPH also showed that shunt surgery led to improvements in symptoms, activities of daily living, and supplementary test results, including results of the Timed Up and Go (TUG) test^7^ for gait disturbance and Mini-Mental State Examination (MMSE)^8^ for dementia.

In developed countries, given the increasing older adults, long-term care (LTC) has become a major social issue^9–11^. Although shunt surgery is effective for iNPH^12,13^, it is not known whether symptomatic improvements afterwards can reduce the LTC burden (LTCB). In this study, we determined whether the LP shunt was effective in decreasing LTCB, using data from the SIN-2, a multicenter study, and implementing repeated measurements. LTCB was assessed for disability and dementia. Another important issue was the degree of variability among the patients and among hospitals in this study. For this purpose, a generalized linear mixed model (GLMM) was applied to assess the treatment effects in entirety (fixed variables) and individual variabilities of treatment effects among patients and among hospitals (random variables) simultaneously^14^.

## Methods

### Participants

**Figure 1:**
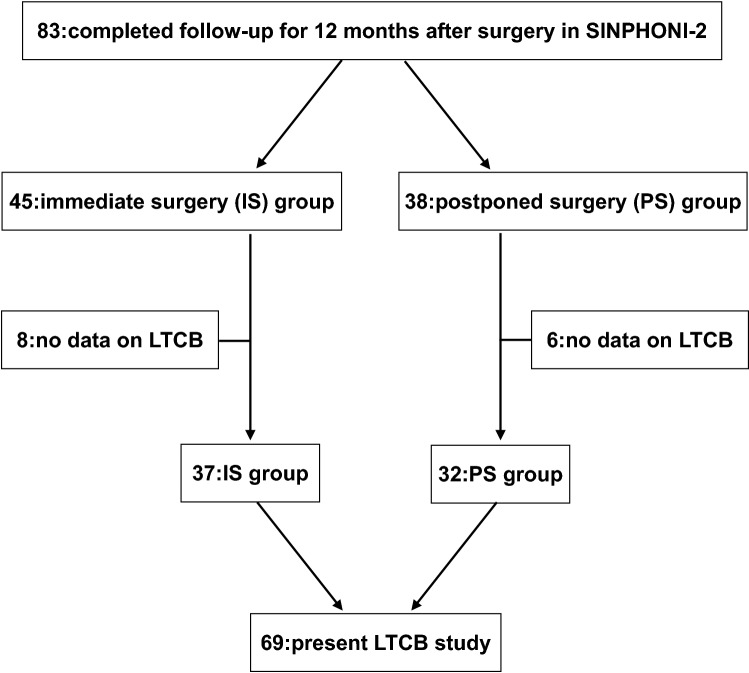
Flowchart of patient’s selection on present LTCB study. *SINPONI-2* study of idiopathic normal pressure hydrocephalus on neurological improvement-2, *LTCB* long-term care burden.

SINPHONI-2 (SIN2) was an open-label randomized trial (UMIN-CTR: UMIN000002730) that followed the Guidelines for Good Clinical Practice and adhered to the principles of the Declaration of Helsinki (2002) of the World Medical Association. The study protocol was approved by the Tohoku University Hospital Ethics Committee and approved by the institutional ethics committee at each site (Rakuwakai Otowa Hospital, Jyuntendo University Hospital, Noto General Hospital, Osaka Medical College, Nishinomiya Kyoritu Hospital, Hamamatsu Medical Centre, Shinko Hospital, Osaka University Hospital, Tokyo Kyosai Hospital, Yokohama Minami Kyosai Hospital, Okayama University Hospital, Kumamoto Takumadai Rehabilitation Hospital, Atsuchi Neurosurgical Hospital, Tama-Hokubu Medical Center, Kanazawa University Hospital, Tama-Nanbu Regional Hospital, Dohtoh Neurosurgical Hospital, Megumino Hospital and Tsudanuma Central General Hospital). Written informed consent was obtained from all patients or their representatives. All clinical and radiological data were prospectively recorded in an independent protocol compliance center via a web-based case report system. The details of the participants, definitions of iNPH, protocol compliance, and data collection (including data acquisition and management) have been described in previous publications^5,6^. In brief, 102 candidates who were diagnosed with possible iNPH according to the second edition of the Japanese iNPH guidelines^12^ were recruited from 20 Japanese centers between March 2010 and October 2011. The inclusion criteria for this study were as follows: patients aged 60–85 years at entry, the presence of one or more symptoms (such as, gait disturbance, cognitive impairment, and urinary disturbance) based on the iNPH grading scale (iNPH: GS) within 3 months before the provision of consent, and ventriculomegaly with an Evans’ index of > 0.3, concurrent with narrow sulci at high convexity and enlarged Sylvian fissure observed on computed tomography or magnetic resonance imaging. The following patients were excluded from this study: patients with diagnosed with secondary hydrocephalus that occurred after subarachnoid hemorrhage, meningitis, head trauma, congenital hydrocephalus, or aqueductal stenosis; patients with CSF pressure of ≥ 20 cmH_2_O; patients with complications of severe disuse muscle atrophy; and psychiatric disorders or other neurological diseases. According to the inclusion and exclusion criteria, 93 patients were registered and randomly assigned to the immediate surgery (IS) or 3-month-postponed surgery (PS) groups (Supple Fig. 1). After randomization, all patients in the IS group underwent lumboperitoneal (LP) shunt surgery using a Codman-Hakim programmable valve with a SiphonGuard (Codman Neuro-DePuy Synthes, Raynham, MA, USA). In the PS group, all patients underwent LP 3 months after registration; during the 3-months period, the patients in the were instructed to perform physical tasks.

The SIN2 design consisted of two parts: (1) assessing the effect of 3-month delay in shunt surgery as a randomised study and (2) assessing the effect of LP shunt as an observational study (Supple Fig. 1). The present study focuses on the latter.

In SIN2, 83 patients were followed up for 12 months after surgery and their data were reported as per-protocol analysis^5,6^. Of the 83 patients included, 45 patients were the IS group and 38 patients were the PS group. The LTCB data for eight patients in the IS group and six patients in the PS group could not be obtained. Thus, 69 patients were finally enrolled in this study: 37 in the former and 32 in the latter (Fig. 1). While 20 hospitals participated in SIN2, only sixteen hospitals participated in this LTCB study (number of enrolled patients ranged from 1 to 17; median was 4).

In the PS group, the preoperative data were recorded 3 months after the registration.

### Assessment of iNPH symptoms and LTCB

Clinical symptoms were evaluated by neurologists, psychiatrists, neuropsychologists, and/or physical therapists who were not in contact with the neurosurgeons performing the LP shunt surgery.

The modified Rankin scale (mRS)^15^ was used to assess the activities of daily living. Gait, cognition, and urinary incontinence were assessed using GS gait, GS cognition, and GS urination items, respectively, on the iNPHGS^5,6^. LTCB for the older adults was assessed based on physical disability and dementia using the Independence Level in Long-Term Care Insurance System, which have been operational in Japan since 2000^16,17^. The severity of disability was originally divided into five major levels with a total of nine grades. A one-grade improvement in LTCB disability was statistically significant^18,19^. However, as the SIN2 did not include patients in the bedridden state, disability was classified into seven levels on the LTCB disability scale (Supple Table 1). The severity of dementia was originally divided originally subcategorized into six major levels, with a total of eight grades. Since, the SIN2 did not include patients with a marked degree of dementia with or without psychomotor symptoms, dementia on the LTCB dementia scale was classified into seven grades (Supple Table 2). The LTCB scales in Japan mainly reflect caregiver burden rather than symptom severity.

### Statistical analysis

All statistical analyses were performed using the R software^20^. Statistical significance was set at p < 0.05. The mean and standard deviation (SD) values for age at entry and the continuous variables were compared using the parametric *t* test. The categorical data were analyzed using the chi-square test. In this study, we applied GLMM as a statistical model. It includes a combination of fixed and random effects as the predictor variables^14^. The fixed effects represent the average treatment effects in all the patients, and random effects represent the individual treatment variabilities of the patients and hospitals. It is also an extension of the linear mixed model, for non-normal data^21^. GLMM is robust for missing data; therefore, listwise deletion of data was no performed^21^. While repeated measures analysis of variance treats time as a categorical variable, GLMM treats time as either a categorical or continuous variable^21^. Since the present data are repeatedly measured data with several of them missing (one at 3 months and three at 6 months; all were the IS group), and non-normal data, as shown by Shapiro’s test^22^, GLMM is useful. We also treated time as continuous.

The *glmer* function in the “lme4” package of R foundation was used to perform GLMM^23,24^. The Poisson family was used with the “log” link. All responder variables (LTC disability, LTC dementia, mRS, GS gait, GS cognition, and GS urination) were regarded as continuous variables. To investigate the variability in the grades of patients and hospitals, we used patients (69 patients) and hospitals (16 hospitals) as random intercept variables. Six fixed variables were selected: time, age, group, sex, TUG test and MMSE. The “Group” and “Sex” were set as categorical variables, and “Time”, “Age”, “TUG” and “MMSE” were set as continuous variables. “Time” was the variable of high interest. “Age,” “Group”, and “Sex” were selected as variables of basic interest. “TUG” and “MMSE” were selected as representatives of motor and cognitive functions. All continuous variables were standardized. Statistical models were built using a single fixed variable and two random intercept variables. Changes in the fixed variables were visualized using the “effects” package^25^. The 95% confidence intervals for fixed variables were derived using the *glmer* function. The intervals for random variables were plotted using the “lattice” package^26^. Although testing the significance of random variables is controversial, we assessed the significance using the 95% profiled confidence interval, which excluded zero^27,28^. When the SD of a random variable was estimated to be zero or near zero, the *glmer* provided a warning of a singular fit. In this instance, a random variable with zero or near-zero values was removed from the model and computed again. When two random variables had zero or near-zero values, they were removed and computation with a generalised linear model (GLM) was applied. When the program provided warning of the non-convergence of the model, the same protocol was applied. Finally, to confirm the significance of the random variables, comparisons between models with (GLMM) and without (GLM) random variables were performed. The Akaike information criterion (AIC) was used as a measure for model selection^29^.

## Results

**Figure 2:**
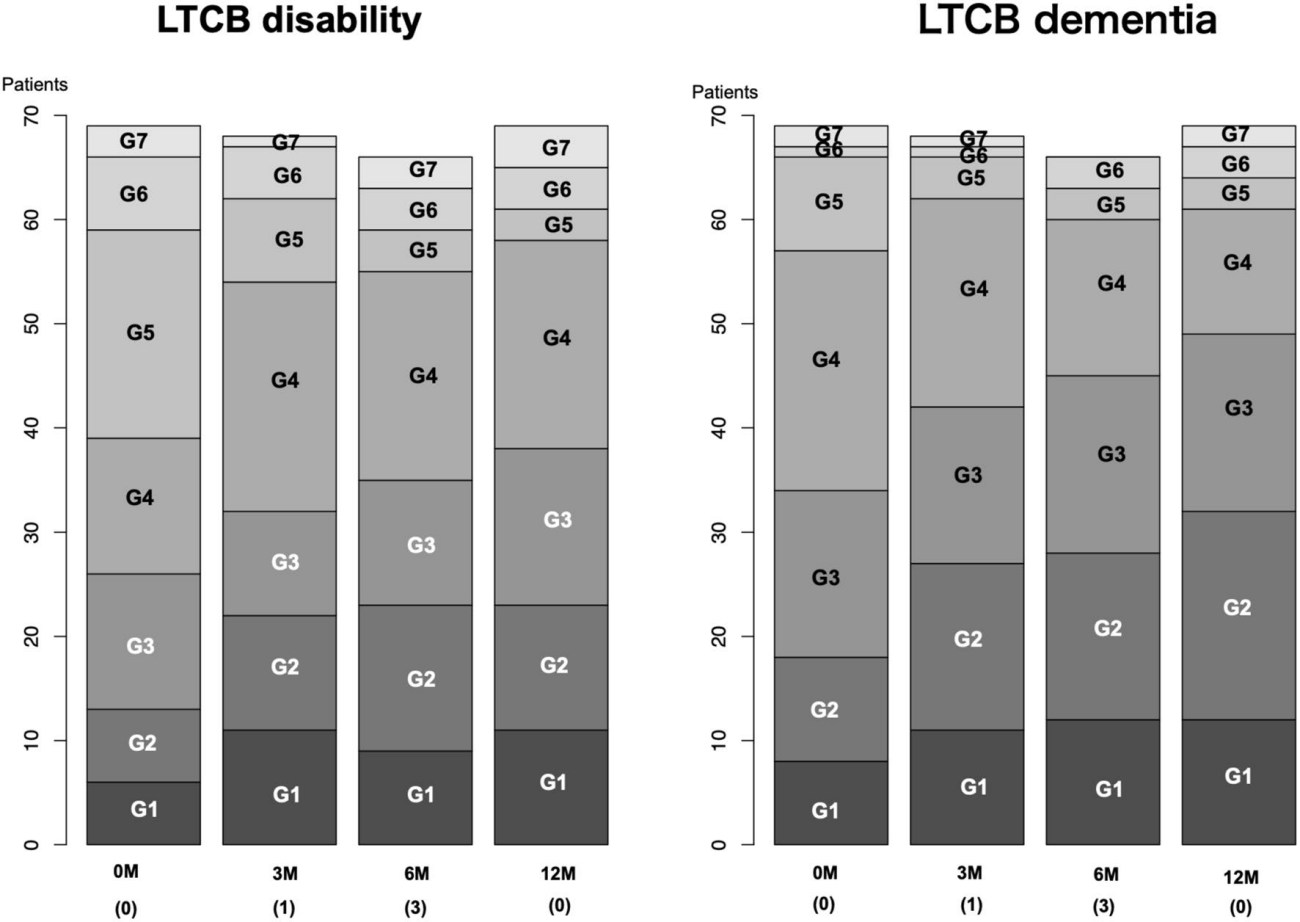
Sequential changes in LTCB grades over 12 months after surgery Data of IS and PS groups were combined. The X-axis indicates four assessment periods (parentheses indicate the number of missing data). Y-axis indicates grades on LTCB scales on disability (left) and dementia (right). Frequencies of low grades (G1, 2, 3) increased over 12 months after surgery for both LTCB disability and dementia.

The clinical characteristics of the patients in the SIN2 (83 patients) and the present LTCB study (69 patients) are summarized in Table 1. There were no significant differences between the SIN2 and LTCB groups. The LTCB consisted of IS and PS groups. The comparison of the two groups revealed no statistical differences, except for sex and urinary disturbance. Sequential changes in the number of patients for each LTCB grade were plotted for both disability and dementia (Fig. 2); the number of patients with low LTCB grades (grades 1 to 3 for disability and grades 1 and 2 for dementia) increased with time. The changes of LTCB disability and dementia grades at 12 months post-surgery (one grade improvement or more) were 53.6% and 49.3%, respectively. Twelve severe adverse events (SAEs) were observed in 11 patients: brain infarcts (n = 4), subdural effusions (n = 3), tube migration (n = 3), tube rupture (n = 1), and spinal fractures (n = 1). Worse LTCB grades due to infarction were observed in two patients. Subdural effusion and shunt tube-related complications were not related to worse LTCB grades.

**Table1:** Clinical characteristics of patients.

	SIN2	Present study	p	Immediate group	Postponed group	p
Cases	83	69		37	32	
Age (mean ± SD)	76.4 ± 4.7	76.1 ± 4.9	NS	76.2 ± 5.2	76.0 ± 5.2	NS
Sex (male %)	54.2	55.1	NS	37.8	75.0	< 0.05*
Onset: gait (%)	78.3	75.4	NS	67.6	83.9	NS
Onset: cognition (%)	44.6	49.3	NS	54.0	48.4	NS
Onset: urination (%)	25.3	29.0	NS	27.0	29.0	NS
mRS (≥ G3) (%)	62.7	58.0	NS	54.1	62.5	NS
GS gait (≥ G3) (%)	47.0	46.4	NS	45.9	46.9	NS
GS cognition (≥ G3) (%)	47.0	50.7	NS	48.6	53.1	NS
GS urination (≥ G3) (%)	28.9	27.5	NS	18.9	37.5	< 0.001*

*G* grade, *GS* iNPH grading scale, *NS* statistically not significant, *p* probability, *SD* standard deviation.

*Statistically significant.

GLMM analysis enabled the simultaneous assessment of both fixed and random effects. The effects of a single fixed variable with two random effects on LTCB disability and dementia are presented in Table 2. For comparison, the same procedure was applied to mRS, GS gait, GS cognition, and GS urination (Tables 2 and 3). In the model for LTCB disability, the SD of individual patients as a random variable ranged from 0.178 to 0.220, and that of the hospital ranged from 0.156 to 0.303. The SD of the random variable (patient individual) was zero (near zero) in two responders (MMSE and FAB), with warnings of a singular fit. To avoid a singular fit, GLMM was reapplied to the model with a single fixed variable and a single random variable (hospital only). Thus, the statistical significance of the fixed variable was shown for all responders but for “Group” and “Sex”. Similar findings were observed for patients with LTCB dementia. One exception was the predictor of “Time,” where statistical significance was not observed. For mRS, both random variables in “TUG” were zero. Then, GLM was applied. For GS urination, most of the models were non-convergent; however, after the removal of the random variable(s), the models using GLM (not GLMM) showed convergence. Other responders to mRS, GS gait, GS cognition, and GS urination showed the same results as those for LTCB disability.

**Table 2:** Results in GLMM (1): LTCB disability, LTCB dementia and mRS.

Responder	FV	AIC	RV: patient	RV: hospital	FV: estimate	CI (upper/lower)	Significance
LTCB disability	Time	1000.66	0.220	0.303	− 0.065	− 0.130/− 0.001	*
	Age	994.75	0.178	0.283	0.143	0.058/0.231	*
	Group	1003.9	0.218	0.290	0.091	− 0.082/0.268	NS
	Sex	1003.83	0.213	0.307	− 0.091	− 0.270/0.089	NS
	TUG	980.02	0.179	0.260	0.104	0.040/0.162	*
	MMSE	944.59	0	0.172	− 0.254	− 0.312/− 0.189	*
LTCB dementia	Time	939.88	0.264	0.252	− 0.049	− 0.118/0.020	NS
	Age	934.98	0.239	0.230	0.131	0.034/0.232	*
	Group	939.63	0.263	0.226	0.146	− 0.048/0.346	NS
	Sex	941.16	0.259	0.256	− 0.082	− 0.281/0.120	NS
	TUG	920.17	0.241	0.215	0.089	0.015/0.157	*
	MMSE	869	0	0.088	− 0.306	− 0.367/− 0.244	*
mRS	Time	856.60	0.136	0.137	− 0.109	− 0.190/− 0.029	*
	Age	856.34	0.088	0.129	0.122	0.035/0.212	*
	Group	861.14	0.106	0.141	0.139	− 0.032/0.308	NS
	Sex	862.83	0.128	0.139	− 0.085	− 0.263/0.093	NS
	TUG	817.3	0	0	0.144	0.088/0.195	*
	MMSE	797.48	0	0.035	− 0.265	− 0.337/− 0.192	*

*AIC* Akaike Information Criteria, *CI* 95% confidence intervals, *dem* dementia, *FV* fixed variable, *GLMM* generalized linear mixed model, *LTCB* long-term care burden, *MMSE* minimental state examination, *mRS* modified Rankin scale, *RV* random variable, *TUG* timed up and go test. *Statistically significant.

**Table 3:** Results in GLMM (2): GS gait, GS cognition and GS urination.

Responder	FV	AIC	RV: patient	RV: hospital	FV: estimate	CI (lower/upper)	Significance
GS gait	Time	819.81	0.248	0.161	− 0.140	− 0.233/− 0.049	*
	Age	821.42	0.209	0.148	0.155	0.045/0.269	*
	Group	826.61	0.221	0.173	0.167	− 0.048/0.380	NS
	Sex	828.55	0.237	0.175	0.073	− 0.157/0.298	NS
	TUG	786.3	0	0	0.165	0.105/0.219	*
	MMSE	797.49	0.178	0.128	− 0.198	− 0.299/− 0.101	*
GS cognition	Time	831.02	0.276	0.212	− 0.094	− 0.181/− 0.007	*
	Age	832.05	0.263	0.183	0.109	− 0.005/0.220	NS
	Group	939.63	0.263	0.226	0.146	− 0.111/0.334	NS
	Sex	833.74	0.259	0.225	− 0.154	− 0.382/0.074	NS
	TUG	806.61	0.218	0.185	0.131	0.051/0.205	*
	MMSE	769.89	0	0.018	− 0.337	− 0.413/− 0.256	*
GS urination	Time	764.58	0.529	nc	− 0.142	− 0.247/− 0.039	*
	Age	786.5	nc	0	0.228	0.123/0.334	*
	Group	803.7	nc	nc	0.119	− 0.081/0.319	NS
	Sex	801.6	nc	0	− 0.188	− 0.388/0.012	NS
	TUG	742.8	nc	nc	0.166	0.098/0.226	*
	MMSE	726.31	0.348	0	− 0.368	− 0.494/− 0.246	*

*AIC* Akaike Information Criteria, *CI* 95% confidence intervals, *FV* fixed variable, *GLMM* generalized linear mixed model, *GS* iNPH grading scale, *MMSE* minimental state examination, *RV* random variable, *TUG* timed up and go test.

*Statistically significant.

**Figure 3:**
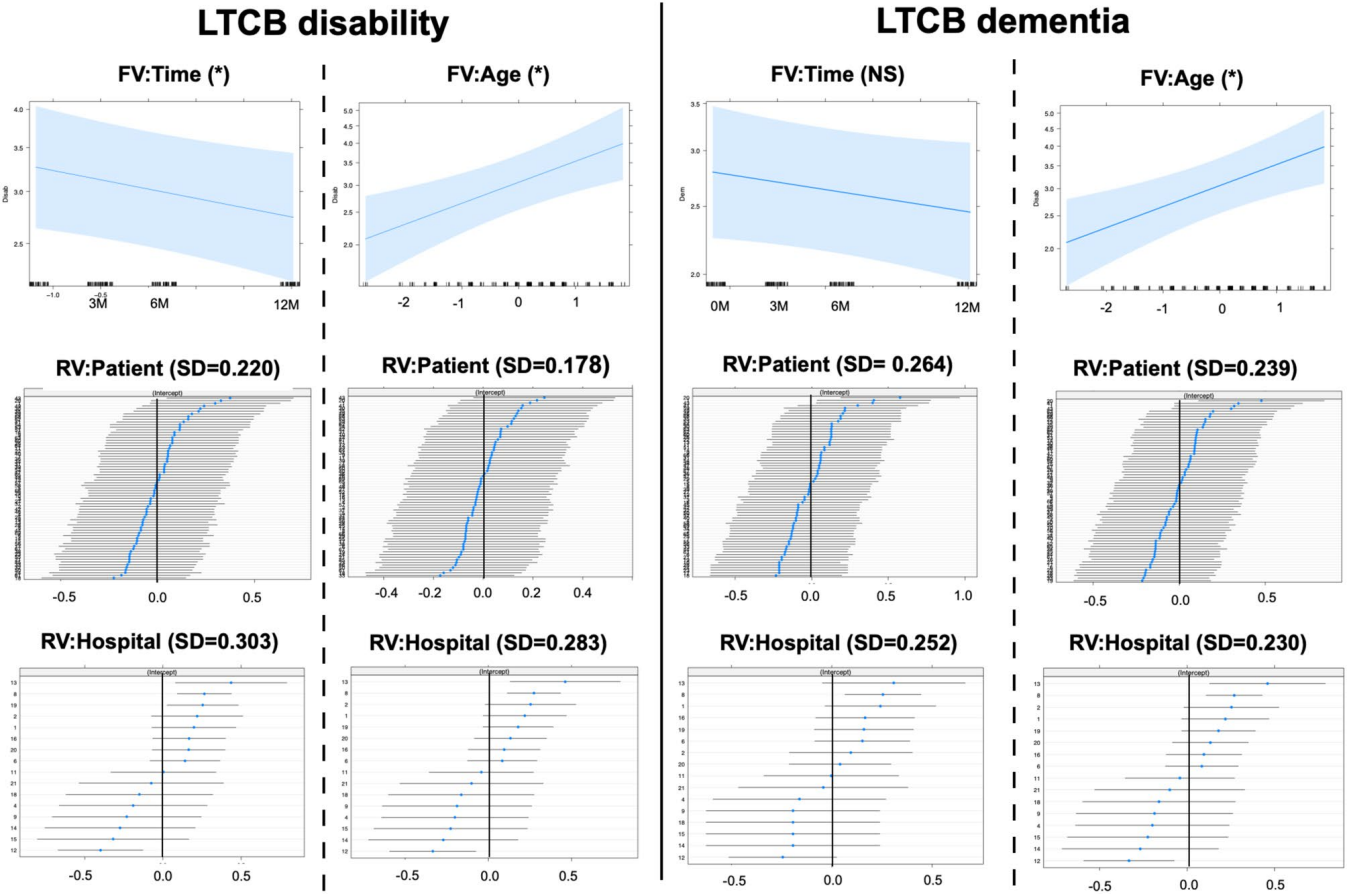
Changes in LTCB grades stratified by Time and Age. As time progressed (upper left), the LTCB grades decreased, but statistical significance was observed only for disability. The shaded area represents a pointwise confidence band for the fitted values. LTCB grades increased with age (upper right). The confidence intervals in random variables (patient individuals: middle, hospitals: lower) are shown. Some specified hospitals (lower) are below or above zero.

**Figure 4:**
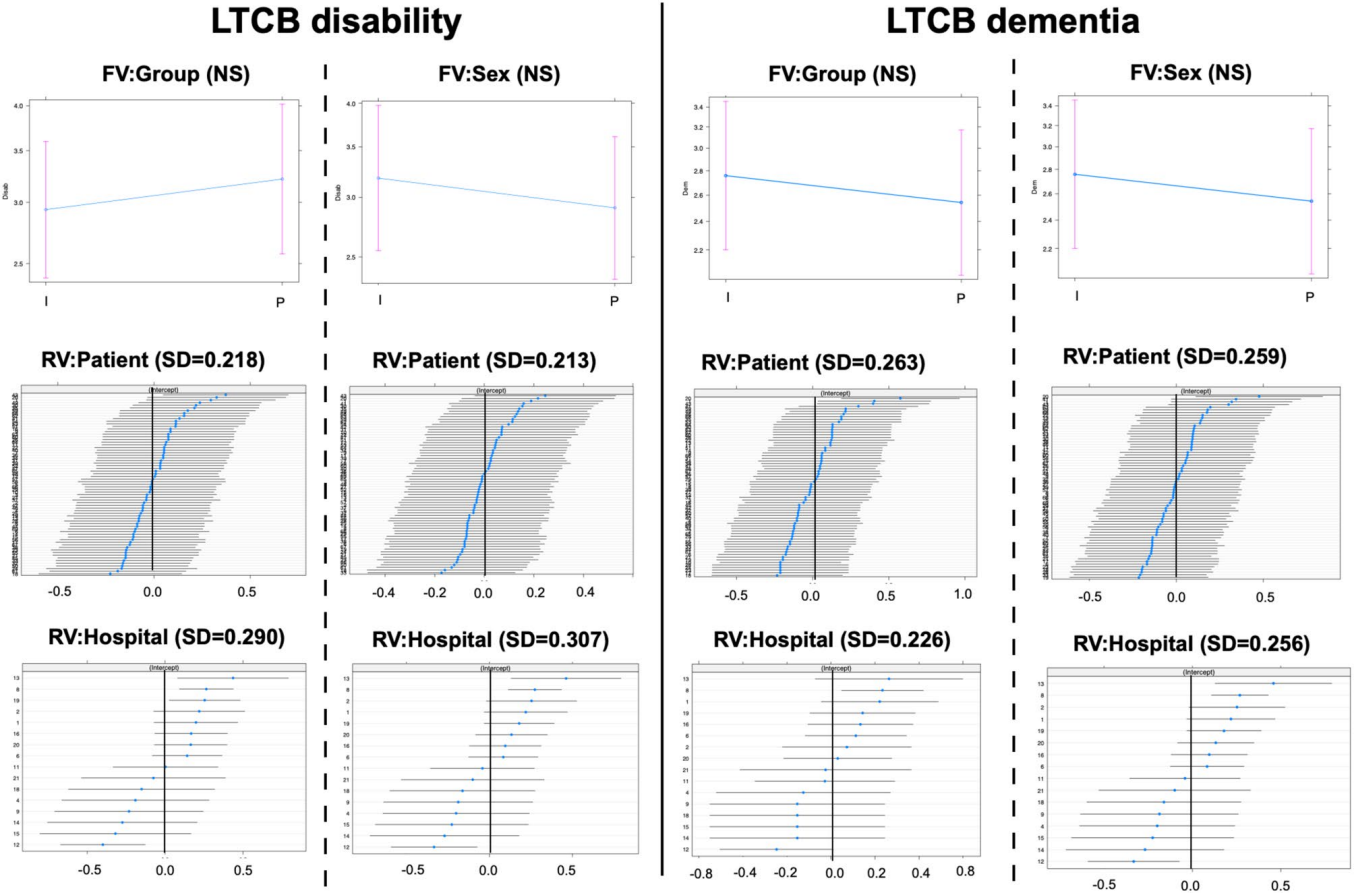
Changes in LTCB grades stratified by Group and Sex. There were no significant differences in LTCB grades (disability: left, dementia: right) between the IS and PS groups and between men and women. Confidence intervals of random variables (patient: middle, hospital: lower) showed that most patients were at zero and some specific hospitals were frequently below or above zero.

**Figure 5:**
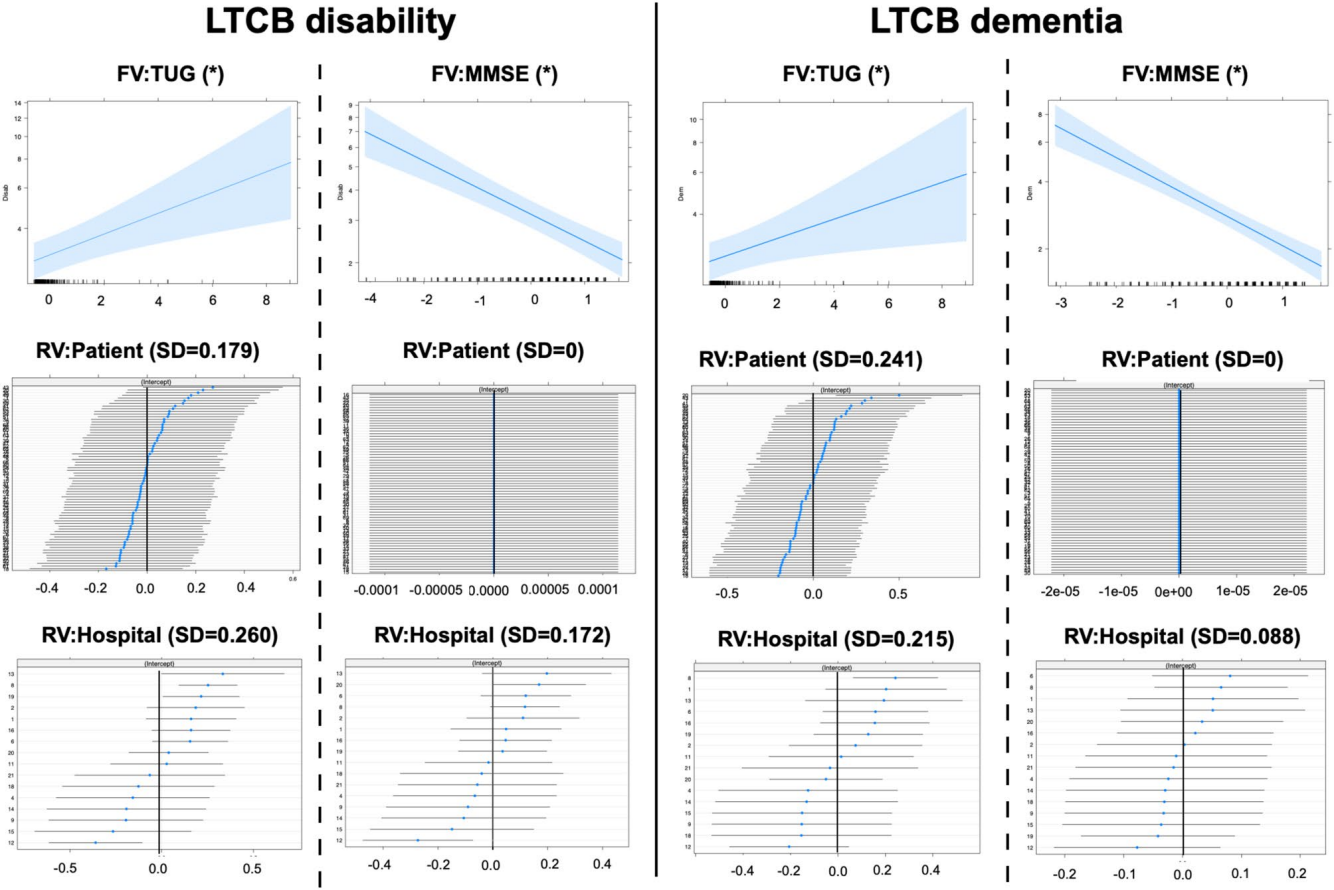
Changes in LTCB grades in TUG and MMSE. As the LTCB grades (disability: left, dementia: right) increased, TUG (upper left) increased and MMSE (upper right) decreased at statistically significant levels. The shaded area represents a pointwise confidence interval for the fitted values. Confidence intervals of random variables (middle and lower) revealed that most patients were at zero and some specific hospitals were below or above zero.

The estimates of the fixed variables for LTCB disability and dementia are plotted in Figs. 3, 4 and 5 (upper panel). As time progressed, the LTCB disability grades decreased with time (Fig. 3). Although the LTCB dementia grades decreased in the same fashion, the changes were not statistically significant. As age increased, the grades of both LTCB disability and dementia increased. Regarding group and sex, no statistical significance was observed for either LTCB disability or dementia (Fig. 4). High LTCB disability grades were associated with increased TUG test scores (Fig. 5) and decreased MMSE scores. The 95% confidence intervals of the MMSE were the narrowest for all fixed continuous variables. For random variables, the 95% confidence intervals for individuals were plotted (Figs. 3,4 and 5; patient, middle; hospital, lowest). The confidence intervals included zero for most of the patients with all responders, and a few patients showed values below or above zero. For hospitals, the same findings were observed, however, some hospitals often showed values above or below zero. Finally, the models with (GLMM) and without (GLM) random effects were compared (Supple Table 3). There were statistically significant differences in all models, except one, and the AICs were lower in the GLMM. The MMSE model in the LTCB showed no significant differences between the GLM and GLMM. Thus, the incorporation of random variables in the model showed an improved fit in almost all models.

## Discussion

This study aimed to assess whether LTCB of patients with iNPH could be improved after CSF shunt surgery, and whether there were differences among individual patients and among hospitals in this study. The present study revealed that LP shunt surgery was effective in improving LTCB disability grades but not LTCB dementia grades within 12 months of follow-up. Age was an important factor underlying the aggravation of LTCB. There were no statistical differences between the IS and PS groups and between male and female patients. The TUG and MMSE scores correlated well with LTCB grades. The incorporation of random variables into the model (GLMM) led to an improved fit. Variability in LTCB grades was more often observed between hospitals than among individual patients.

The present study focused on the effect of surgery on the LTCB aspects of disability and, particularly dementia. Our previous report did not focus on LTCB^5^. Many studies have indicated that CSF shunt surgery is effective in patients with iNPH^1–4^. However, most of them aimed to examine improvement in symptoms or supplementary examination results, but not LTCB. Notably, Kazui et al.^30^ reported an improvement in caregiver burden after surgery in patients with iNPH in their prospective study. Israelsson et al.^31^ found improvements in the quality of life assessed using the EuroCol 5-dimensions instrument in a Swedish population, and showed that the quality of life remained improved in shunted patients after iNPH at a mean follow-up period of 21 months, although the patients did not attain the same quality of life as the regular population. Since most patients with iNPH are in their seventies or eighties and have some degree of dementia, they are candidates for LTC. The LTC insurance system in Japan requires candidates to be assessed by a doctor for disability and dementia before they can receive it. In this system, family doctors assess the severity of LTCB disability and dementia (Tables 1 and 2). A high correlation between LTCB disability and functional independence measures has been reported^18^. The LTCB of dementia scale mainly reflects dementia-related ADL, with a small influence on the behavioural and psychological symptoms of dementia^19^. In this sense, the LTCB disability and dementia scales in Japan mainly reflect caregiver burden, rather than the symptomatic severity of disability and dementia. Since LTCB can differ with on socioeconomic and cultural backgrounds, individual studies are necessary.

In this study, the effect of shunt surgery on LTCB disability and dementia was evaluated, in parallel with the assessment of ADL using the mRS and assessments of major symptoms using the iNPHGS. With time, the changes in LTCB disability grades, mRS, GS gait, GS cognition, and GS urination showed statistically significant improvements. This is consistent with the SIN2 results. Since gait disturbance in iNPH is the most responsive symptom after shunt surgery, the result would reflect improvement in LTCB disability.

The improvement in LTCB dementia grades was not statistically significant, which was in contrast with the improvement in the MMSE scores in SIN2. This is consistent with the general impression of less improvement in dementia grades than in gait and urinary disturbances. However, the LTCB dementia grades showed a gradual decrease, indicating the need for the further studies to improve dementia grades. Early shunt surgery may be a good option for patients with iNPH with cognitive function within the range of mild cognitive impairment.

In this study, we applied GLMM as a statistical model. Diaz^14^ highlighted that mixed models (linear mixed model and GLMM) are valuable in personalised medicine, which focuses on the analysis of individuals rather than the average effects of treatment. Using GLMM, we found that the 95% confidence intervals of some hospitals were above or below zero more often, in contrast with the patients. This indicated that there were differences in the LTCB grades of the hospitals, particularly in a few hospitals showing out of 95% CI. Since there were few patients per hospital (median: 4 patients), the statistical power may have been low. However, the differences in the LTCB grades across the hospitals may be attributed to the differences in the assessments. Another possibility on the differences in the LTCB differences is a difference in surgical volume. Hospitals with large surgical cases can show better results. However, since SIN2 had been performed during the developing stage of the LP shunt surgery for iNPH on the surgical technique, its possibility is low.

This study had some limitations. The assessments using the LTCB scales were optional in the SIN2 protocol, hence, some hospitals did not report them. Thus, LTCB data from eight patients in the IS group and six patients in the PS group were not obtained. As we were interested in the effects of shunt surgery on LTCB, a new dataset was generated from the original. The datasets were comparable; however, some differences related to sex and GS urination were noted. This may have led to different interpretations of the data. Although the data for the LTCB scales were not continuous in a strict sense, we regarded them as continuous variables, as in the mRS or GS data. The analysis of comorbidities was not included in the study for avoiding complexity. However, comorbidities are important factors that affect the outcomes. Particularly, the comorbidity of Alzheimer’s disease is important in patients with iNPH. Further prospective studies of iNPH with long-term follow-up are necessary.

## Conclusions

LP shunt surgery significantly improved the LTCB of disability over 12 months after surgery, but not the LTCB of dementia. Differences were more often observed among hospitals than among individual patients. Further efforts are needed to improve dementia in patients with iNPH. Furthermore, the incorporation of random variables, such as hospitals, is important for the analysis of data from multicenter studies.

## Supplementary Information

Supplementary Dataset 1.

Supplementary Dataset 2.

Supplementary Table legends.

Supplementary Tables.

Supplementary Figure S1.

Supplementary Figures S1 legend.

## Data Availability

The dataset supporting this article is included with the supplementary information.
