# Supplementary material for: Improvement in the long-term care burden after surgical treatment of patients with idiopathic normal pressure hydrocephalus: a supplementary study
Source: Sci Rep. 2021 Jun 3;11:11732. doi: 10.1038/s41598-021-90911-2 (PMC8175749; doi:10.1038/s41598-021-90911-2)
Supplement: Supplementary file 3 — Supplementary Table legends. [file 41598_2021_90911_MOESM3_ESM.pdf]

Supplementary information

Improvement in the long-term care burden after surgical treatment of patients with idiopathic normal pressure hydrocephalus: a supplementary study

Masatsune Ishikawa[1,2*], Shigeki Yamada[3, 2], Masakazu Miyajima[4], Hiroaki Kazui[5], and Etsuro Mori[6]

[1]Rakuwa Villa Ilios, Kyoto, Kyoto, Japan

[2]Normal pressure hydrocephalus Centre, Otowa Hospital, Kyoto, Kyoto, Japan

[3]Department of Neurosurgery, Shiga University of Medical Science, Otsu, Shiga, Japan

[4]Department of Neurosurgery, Juntendo Tokyo Koto Geriatric Medical Center, Kotoku, Tokyo, Japan

[5]Department of Neuropsychiatry, Kochi Medical School, Kochi University, Nankoku, Kochi, Japan

[6]Department of Behavioral Neurology and Neuropsychiatry, Osaka University United Graduate, Toyonaka, Osaka, Japan

Supplementary Table legends:

1. **Supplementary Table 1 [SuppleTable1]:** Independence level in disability in long-term care insurance system in Japan (LTCB disability)

2. **Supplementary Table 2 [SuppleTable2]:** Independence level in dementia in long-term care insurance system in Japan (LTCB dementia)

3. **Supplementary Table 3 [SuppleTable3]:** Model comparison between GLM and GLMM

4. **Supplementary data file 1 [SuppleData1]:** LTCB data in a wide format

5. **Supplementary data file 2 [SuppleData2]:** LTCB data in a long format
