# Supplementary material for: Improvement in the long-term care burden after surgical treatment of patients with idiopathic normal pressure hydrocephalus: a supplementary study
Source: Sci Rep. 2021 Jun 3;11:11732. doi: 10.1038/s41598-021-90911-2 (PMC8175749; doi:10.1038/s41598-021-90911-2)
Supplement: Supplementary file 4 — Supplementary Tables. [file 41598_2021_90911_MOESM4_ESM.docx]

**SuppleTable1: Independence Level in Disability in Long-Term Care Insurance System in Japan (LTCB disability)**

| **Original** | **Present** | **Content** |
| --- | --- | --- |
| **In** | **G1** | **independent** |
| **J** |  | **has some disability, but is almost completely independent in daily activities and able to go outside w/o support** |
| **J1** | **G2** | **able to go outside and use public transportation** |
| **J2** | **G3** | **able to go outside to the neighborhood** |
| **A** |  | **is mostly independent in daily activities at home, but not able to go outside w/o support** |
| **A1** | **G4** | **go outside with support and is out of bed in most of the daytime** |
| **A2** | **G5** | **seldom goes outside, and frequently lie in bed in the daytime.** |
| **B** |  | **requires some support in daily activities in the house, and is mostly confined to the bed, but is able to maintain a sitting position.** |
| **B1** | **G6** | **able to transfer to a wheelchair, and able to take meals and toilet out of bed** |
| **B2** | **G7** | **able to transfer to wheelchair w/ support** |
| **C** |  | **is in bed whole day, and requires support for toileting, taking meals and changing clothes.** |
| **C1** |  | **able to turn over in bed w/o support** |
| **C2** |  | **not able to turn over in bed by oneself** |

G: grade

**SuppleTable2: Independence Level in Dementia in Long-Term Care Insurance System in Japan** **(LTCB dementia)**

| **Original** | **Present** | **Content** |
| --- | --- | --- |
| **In** | **G1** | **independent** |
| **I** | **G2** | **has some dementia, but is almost completely independent of daily life in the house and society** |
| **II** |  | **has some difficulties in daily life and communication, but still independent on watching by someone** |
| **IIa** | **G3** | **shows above grade II in the house** |
| **IIb** | **G4** | **shows above grade II out of the house** |
| **III** |  | **has some difficulties in daily life and communication, and needs nursing care** |
| **IIIa** | **G5** | **shows above grade III mainly during day-time** |
| **IIIb** | **G6** | **shows above grade III including night-time** |
| **IV** | **G7** | **has frequent difficulties in daily life and communication, and needs constant nursing care** |
| **M** |  | **has marked psychological or behavioral symptoms, which are necessary for treatment by specialists** |

G:grade

**SuppleTable3: Model comparison between GLM and GLMM**

|  | **LTCB disability** | | **LTCB dementia** | |
| --- | --- | --- | --- | --- |
|  | **AIC (GLM vs. GLMM)** | **p value** | **AIC (GLM vs. GLMM)** | **p value** |
| **Time** | **1037.5 vs. 1000.9** | **<0.001** | **967.3 vs. 939.9** | **<0.001** |
| **Age** | **1019.4 vs. 994.5** | **<0.001** | **955.1 vs. 935.0** | **<0.001** |
| **Group** | **1038.5 vs. 1003.8** | **<0.001** | **964.3 vs. 939.6** | **<0.001** |
| **Sex** | **1040.5 vs. 1003.9** | **<0.001** | **968.3 vs. 941.2** | **<0.001** |
| **TUG** | **1002.1vs. 980.1** | **<0.001** | **940.0 vs. 020.2** | **<0.001** |
| **MMSE** | **948.8 vs. 946.6** | **<0.05** | **869.0vs. 872.4** | **0.623** |

AIC: Akaike information criteria, GLM: generalized linear model, GLMM: generalized linear mixed model, LTCB: long-term care burden, p: probability, MMSE: mini-mental state examination, TUG: timed up and go test
