# Supplementary figures and images for: Improvement in the long-term care burden after surgical treatment of patients with idiopathic normal pressure hydrocephalus: a supplementary study
Source: Sci Rep. 2021 Jun 3;11:11732. doi: 10.1038/s41598-021-90911-2 (PMC8175749; doi:10.1038/s41598-021-90911-2)

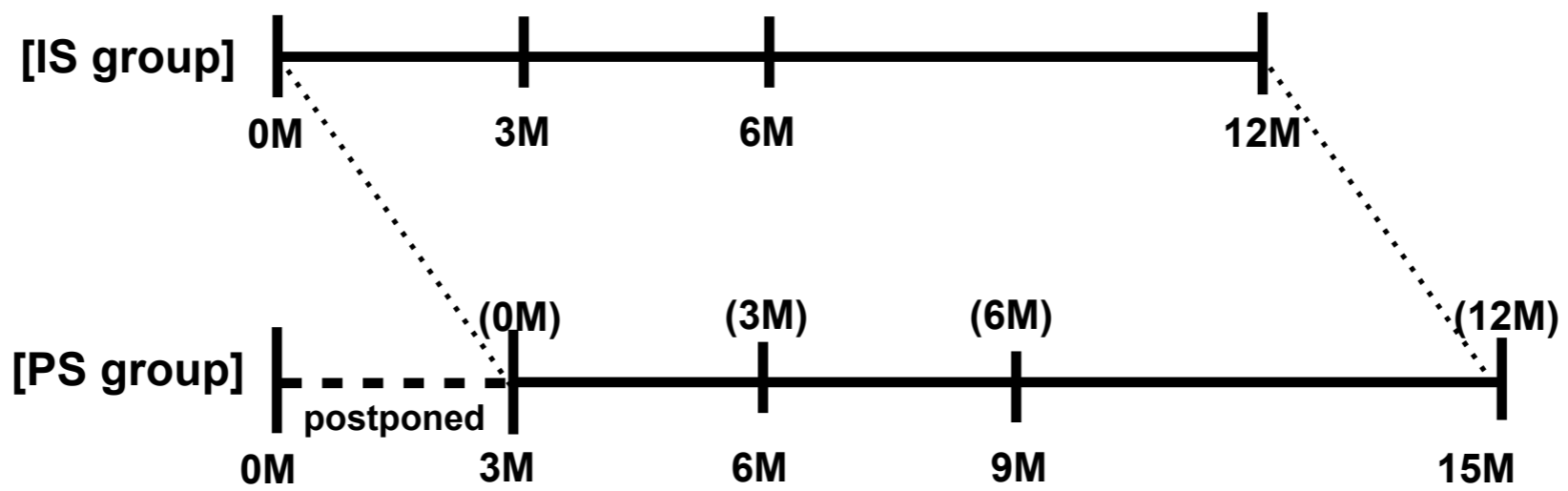

Supplement: Supplementary file 5 — Supplementary Figure S1. [file 41598_2021_90911_MOESM5_ESM.pdf]
