# Supplementary material for: Improvement in the long-term care burden after surgical treatment of patients with idiopathic normal pressure hydrocephalus: a supplementary study
Source: Sci Rep. 2021 Jun 3;11:11732. doi: 10.1038/s41598-021-90911-2 (PMC8175749; doi:10.1038/s41598-021-90911-2)
Supplement: Supplementary file 6 — Supplementary Figures S1 legend. [file 41598_2021_90911_MOESM6_ESM.pdf]

Supplementary figure legend

SuppleFig.1. Study design of the SINPHONI-2

Patients were randomly assigned into the immediate surgery (IS) group and the postponed surgery (IS) group. The IS group was operated within 1 month after the diagnosis of possible iNPH. They were followed up for 12 months, with assessments before surgery and at 3, 6, and 12 months after. In the PS group, the surgery was postponed for 3 months; then, the patients were followed up similar to the IS group.
